# Role of gut microbiota in regulating gastrointestinal dysfunction and motor symptoms in a mouse model of Parkinson’s disease

**DOI:** 10.1080/19490976.2020.1866974

**Published:** 2021-01-18

**Authors:** Yogesh Bhattarai, Jie Si, Meng Pu, Owen A. Ross, Pamela J. McLean, Lisa Till, William Moor, Madhusudan Grover, Karunya K. Kandimalla, Kara G. Margolis, Gianrico Farrugia, Purna C. Kashyap

**Affiliations:** a Division of Gastroenterology and Hepatology, Mayo Clinic, Rochester, MN, USA; b Department of Neuroscience, Mayo Clinic, Jacksonville, FL, USA; c Department of Physiology and Biomedical Engineering, Mayo Clinic, Rochester, MN, USA; d Department of Pharmaceutics, College of Pharmacy, University of Minnesota, Minneapolis, MN, USA; e Division of Pediatric Gastroenterology, Hepatology and Nutrition, Morgan Stanley Children’s Hospital, Columbia University Irving Medical Center, New York, NY, USA

**Keywords:** Microbiota-gut-brain axis, intestinal epithelial barrier, idiopathic Parkinson's disease, gnotobiotic mice, Braak hypothesis

## Abstract

Parkinson’s disease (PD) is a common neurodegenerative disorder characterized primarily by motor and non-motor gastrointestinal (GI) deficits. GI symptoms’ including compromised intestinal barrier function often accompanies altered gut microbiota composition and motor deficits in PD. Therefore, in this study, we set to investigate the role of gut microbiota and epithelial barrier dysfunction on motor symptom generation using a rotenone-induced mouse model of PD. We found that while six weeks of 10 mg/kg of chronic rotenone administration by oral gavage resulted in loss of tyrosine hydroxylase (TH) neurons in both germ-free (GF) and conventionally raised (CR) mice, the decrease in motor strength and coordination was observed only in CR mice. Chronic rotenone treatment did not disrupt intestinal permeability in GF mice but resulted in a significant change in gut microbiota composition and an increase in intestinal permeability in CR mice. These results highlight the potential role of gut microbiota in regulating barrier dysfunction and motor deficits in PD.

## Introduction

Parkinson’s disease (PD) is a progressive neurodegenerative disorder which affects nearly 10 million people worldwide with about a million active cases in the US alone, and nearly 60,000 new cases reported each year.^1,2^ Neuropathologically, PD is characterized by accumulation of alpha-synuclein aggregates and loss of dopaminergic neurons within the substantia nigra pars compacta (SNpc) region of the brain ,^3^ leading to classical motor deficits such as bradykinesia, resting tremor, rigor and postural instability.^4^ In addition to motor deficits, PD patients also develop non-motor deficits including olfactory changes, cognitive decline, and gastrointestinal (GI) symptoms.^5^ These non-motor deficits especially GI symptoms affect a majority (60–80%) of PD patients and can precede the onset of motor symptoms by several years.^6–8^ The high prevalence and early onset of GI deficits in PD have led to several proposed mechanisms underlying the development of PD, including a role for disrupted intestinal permeability, altered immune activation, disrupted GI motility, and alterations in the gut microbiota.^9^

The vast majority of PD cases are idiopathic and possibly caused by a complex interplay of environmental factors and genetic predisposition. While the exposure to pesticides and environmental toxins likely plays a crucial role in the initiation and pathogenesis of idiopathic PD, the majority of studies investigating PD till date are performed in genetic rodent models of PD.^10^ There is a gap in the literature on the role of gut microbiota in determining the impact of environmental toxins in PD. Rotenone is a naturally occurring pesticide that has been previously shown to cause motor dysfunction similar to PD in mice.^11,12^ In this study, we test the effect of rotenone in germ-free (GF) and conventionally raised (CR) mice, with the specific goal of studying a potential impact of gut microbiota and GI dysfunction in the development of motor symptoms in PD.

## Results

### Chronic rotenone administration results in loss of tyrosine hydroxylase motor neurons independent of the gut microbiota

**Figure 1.**
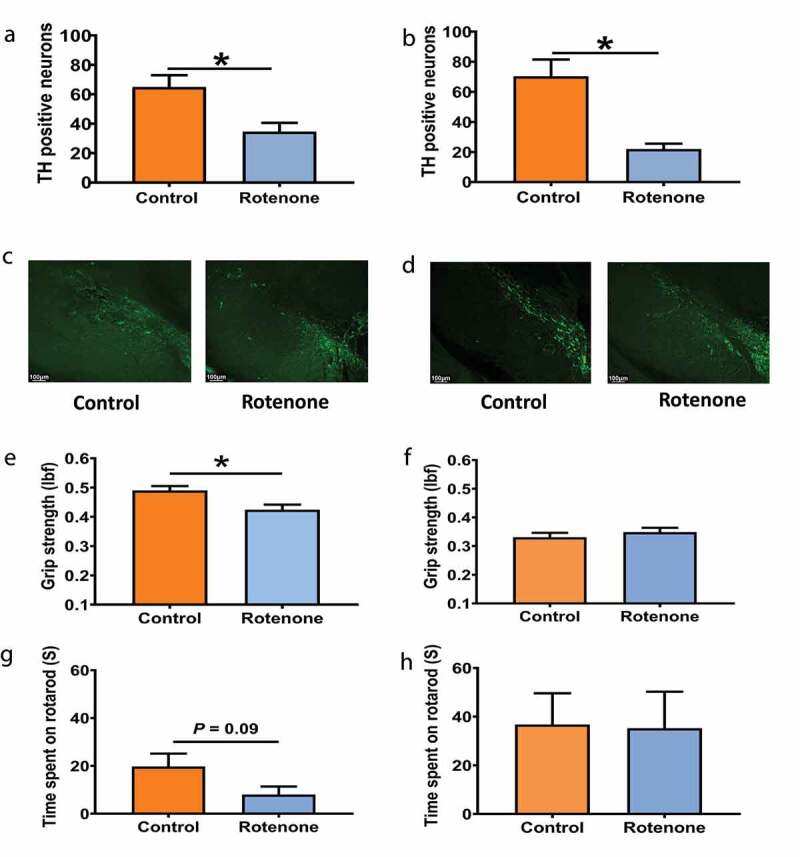
Chronic rotenone administration causes motor dysfunction in CR mice but not in GF mice. Change in TH-neuron number in substantia nigra following treatment with rotenone (10mg/kg) or vehicle with representative images in CR (A, C) and GF mice (B, D). Changes in grip strength and time spent in rotarod in CR mice (E, G) and GF mice (F, H) respectively following treatment with rotenone (10mg/kg) or vehicle. The data are presented as mean ± SEM [unpaired Student's t-test (two-tailed); *P <0.05]

Rotenone administration has been shown to cause TH-neuron loss in a previous study,^13^ hence we quantified TH neuron using IHC in both GF (Figure 1b,d) and CR (Figure 1a,c) mice following rotenone treatment. We found that rotenone administration resulted in significantly greater TH-neuron loss within the substantia nigra region in both GF (70 ± 11.2 vs 22 ± 3.5, n = 4–5, P < .01, Figure 1b) and CR (64.9 ± 8.0 vs 34.6 ± 5.9, n = 4, P < .05, Figure 1a) mice when compared to vehicle-administered control mice (Figure 1a–d).

### The development of motor dysfunction following chronic rotenone administration is dependent on the presence of gut microbiota

To investigate the role of gut microbiota in PD pathogenesis, we examined the effect of chronic rotenone administration on motor function in GF and CR mice. There was a significant decrease in grip strength (0.49 ± 0.015 vs 0.42 ± 0.017, n = 9–10, *P* < .05) and an overall decrease in motor (19.75 ± 5.4 vs 8.0 ± 3.4, n = 4–5, *P* = .09) coordination as measured by rotarod test following administration of rotenone (10 mg/kg) for 6 weeks in CR mice (Figure 1e,g) but not in GF mice (Figure 1f,h) when compared to vehicle-treated mice. This suggests that motor dysfunction following rotenone administration is dependent on the presence of gut microbiota.

### Chronic rotenone administration is associated with disruption in epithelial barrier only in the presence of gut microbiota

**Figure 2.**
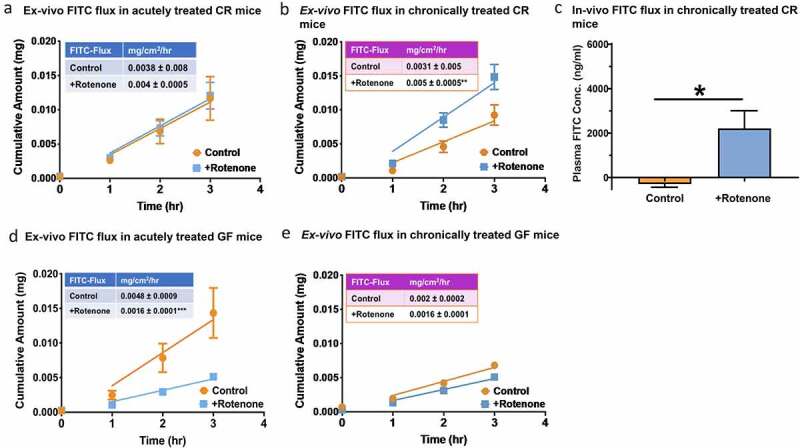
Chronic rotenone administration disrupts epithelial permeability and alters gut microbiota composition in CR mice. The cumulative fluorescein isothiocyanate (FITC) flux across the *ex-vivo* colonic mucosal explants obtained from (A) acutely rotenone and vehicle treated CR mice and (B) chronically rotenone and vehicle treated CR mice. (C) *In-vivo* FITC flux in CR mice subjected to chronic rotenone and vehicle treatment. *Ex-vivo* FITC flux across colonic mucosal explants in (D) acutely rotenone and vehicle treated GF mice and (E) chronically rotenone and vehicle treated GF mice. The data is presented as mean ± SEM. Difference between the slopes are calculated by F-test; in chronically treated CR: DFn = 1, DFd = 20; **P<0.01 and in acutely treated GF: DFn = 1, DFd = 28; **P<0.01. Plasma absorption of orally administered FITC in chronically vehicle control versus rotenone treated CR mice [Student’s t-test (two-tailed); *P <0.05]

Chronic (6 weeks) but not acute (4 hours) rotenone administration in CR mice resulted in a significant increase in *ex-vivo* (proximal colon mucosa-submucosa preparation) 4 kDa FITC-flux (n = 3–4, Figure 2a–b). Chronic rotenone administration (6 weeks) also resulted in a significant increase in in-vivo 4 kDa FITC (n = 3–4, P < .05, Figure 2c) but not creatinine flux (data not shown) compared to vehicle-treated CR mice suggesting disruption of the leak but not the pore pathway. The lack of change with acute rotenone administration in CR mice suggests that the effect of rotenone on intestinal permeability is likely not due to a direct effect of rotenone on the epithelium. Next, to determine if the effect of rotenone on intestinal permeability is dependent on the presence of gut microbiota, we repeated the same experiments in GF mice. We found that chronic rotenone administration in GF mice had no effect on 4 kDa FITC-flux in *ex-vivo* proximal colon mucosa-submucosa preparation. However, in contrast to the findings in CR mice, acute rotenone administration in GF mice significantly decreased 4 kDa FITC-flux in *ex-vivo* proximal colon mucosa-submucosa preparation compared to vehicle-treated control mice (n = 4–5, Figure 2d–e) suggesting reinforcement of epithelial barrier in acutely rotenone treated mice. Together these data suggest that increased intestinal permeability following chronic rotenone administration in CR mice is dependent on the presence of gut microbiota.

### Chronic rotenone administration in CR mice results in changes in gut microbiota composition similar to those observed in PD patients

**Figure 3.**
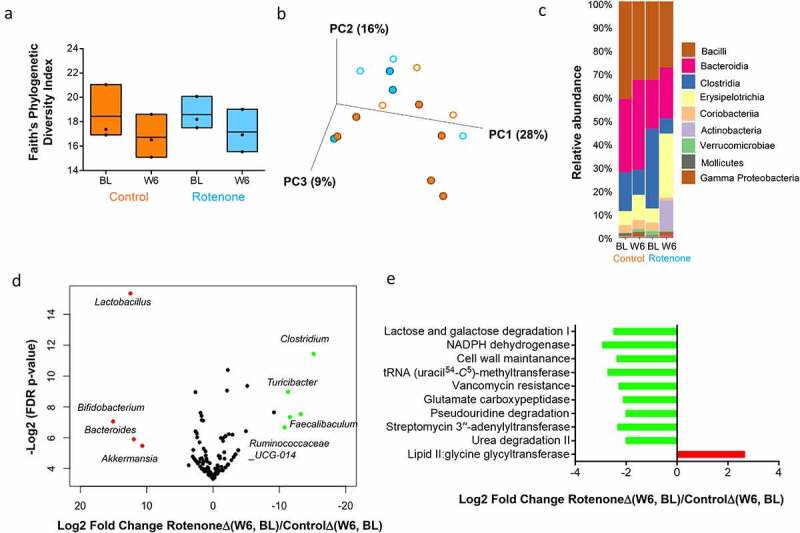
Changes in gut microbiota composition following rotenone or vehicle treatment in CR mice. (a) Change in α-diversity from baseline (BL) to week 6 (W6) following rotenone (blue) or vehicle (orange) treatment in conventionally raised (CR) mice was assessed using Faith’s phylogenetic diversity index. Kruskal–Wallis (pairwise) test, vehicle control: BL vs W6, *q = 0.6*; rotenone: BL vs W6, *q = 0.6*; BL: rotenone vs vehicle control, *q* = 0.7; W6: rotenone vs vehicle control, *q* = 0.7. A comparison of the change in α-diversity from BL to W6 following rotenone [RotenoneΔ(W6, BL)] or vehicle treatment [ControlΔ(W6, BL] shows no statistically significant difference (Wilcoxon Sign-Rank Test) between the treatment groups. (b) Bray Curtis-based PCoA plot (2-dimensional representation of 3-dimensional plot) of gut microbial communities in CR mice treated with vehicle solution (orange; control group) or rotenone solution (blue). BL samples are represented by open circles while W6 samples are represented by filled circles. There was no statistically significant difference in beta diversity at BL (PERMANOVA test; *q* = 0.26) or W6 (PERMANOVA test; *q* = 0.23) between rotenone and vehicle-treated mice. A comparison of the change in beta diversity from BL to W6 following rotenone [RotenoneΔ(W6, BL)] or vehicle treatment [ControlΔ(W6, BL] shows no statistically significant difference (Wilcoxon Sign-Rank Test) between the treatment groups. (c) Bar plot showing changes in relative abundance of different bacterial classes from BL to W6 following treatment of CR mice with rotenone (blue) or vehicle (orange). (d) Volcano plot showing compositional difference in gut microbiota between vehicle (control) and rotenone treated CR mice. Log2-transformed fold change in microbiota composition (RotenoneΔ(W6, BL)/ControlΔ(W6, BL)) is plotted on the x-axis and log-transformed, FDR adjusted *p*-values are plotted on the y-axis. Bacterial genera that are significantly increased [FDR adjusted *P* < .05] following rotenone administration are represented in red while those that are significantly decreased are represented in green. (e) Comparison of the change in predicted microbial metabolic pathways (PICRUSt) FDR<0.25) from BL to W6 following vehicle and rotenone treatment in CR mice [RotenoneΔ(W6, BL)/ControlΔ(W6, BL)]. Pathways (FDR<0.25) that are significantly increased are shown in red while those that are significantly decreased following rotenone administration are shown in green

To investigate the effect of chronic rotenone administration on gut microbiota composition, we characterized changes in gut microbiota composition in fecal samples from CR mice before and after treatment with rotenone or vehicle solution (control group) using 16S rRNA amplicon-based sequencing. There were no significant differences in beta diversity (based on Bray Curtis metric) or alpha diversity (based on Faiths Phylogenetic Diversity) following rotenone treatment when compared to vehicle treatment (Figure 3a,b). There were changes in the relative abundances of multiple bacterial classes after rotenone treatment such as Clostridia which was not seen following vehicle treatment (Figure 3c). We also found rotenone treatment resulted in an increase in the relative abundance of bacterial genera *Lactobacillus, Bifidobacterium, Akkermansia*, and *Bacteroides* spp and a decrease in *Lachnospiraceae, Ruminococcaceae_UCG-014, Turicibacter, Faecalibaculum*, and *Clostridium* spp when comparing changes in bacterial genera from baseline to week 6 in rotenone and vehicle-treated mice (Figure 3d). Interestingly, the microbial changes that we observed in our rotenone-treated mice such as increased *Lactobacillaceae, Bacteroides*, and decreased *Lachnospiraceae* have also been described in human PD studies and have been associated with worsening clinical symptoms, such as higher frequencies of gait disturbances, and postural instability.^14^ We also imputed changes in microbial metabolic pathways from 16S rRNA using the Phylogenetic Investigation of Communities by Reconstruction of Unobserved States Predicted pathway function (PICRUSt2) and found the LipidII: glycine glycyltransferase pathway involved in bacterial peptidoglycan synthesis was significantly upregulated following rotenone treatment when compared to changes following vehicle treatment in control mice (Figure 3e).

## Discussion

In this study, we found that chronic administration of rotenone results in disrupted colonic epithelial permeability and development of motor symptoms only in CR mice, which harbors a complex microbiota, but not in GF mice. Additionally, rotenone did not have an effect on epithelial permeability when colonic tissues from CR mice were acutely exposed to rotenone. Together these data suggest that gut microbiota is not only required but may in fact be the mediators of the effect of environmental toxins on intestinal permeability as well as the development of motor symptoms.

In our study we observed a similar loss of TH neurons within the substantia nigra in both GF and CR mice following rotenone administration. Interestingly however, motor dysfunction was only seen in CR mice. The discordance between TH-neuron loss and motor deficits observed in our study has also been reported in previous studies,^15–18^ which show that the adaptive capacity of the nigrostriatal dopaminergic pathway can compensate for TH neuron loss and help preserve motor function. Our findings would suggest that motor dysfunction in PD is not driven exclusively by TH-neuron loss but is likely a result of the complex interplay of multiple factors. These may include microglia activation, soluble, and insoluble α-synuclein pathologic strains within the substantia nigra and the caudoputamen region of the nigrostriatal pathway,^19^ and modulation of signaling within the basal ganglia structures via bacteria (e.g. *Lactobacillus* and *Bifidobacterium* spp.) derived neurotransmitters like glutamate or gamma aminobutyric acid.^20–24^

Several studies till date have examined gut microbiota alterations in rotenone animal models and often produced varying results. In contrast to the results obtained in our study, Perez-Pardo et al. observed a significant decrease in the relative abundance of *Bifidobacterium* and a corresponding increase in *Ruminococcaceae* in the cecum of a rotenone treated mice.^25^ Johnson and colleagues, on the other hand, reported a significant increase in *Bifidobacterium* and *Lactobacillus* and a corresponding decrease in *Ruminococcaceae* and *Lachnospiraceae* in the small intestine and colon of a rotenone-treated rats.^26^ The variability in microbiota composition among different studies could be a result of several factors including differences in animal species, microbiota sampling sites, lack of standardization in concentration, duration, and route of administration of rotenone, and sample processing and sequencing.^9^ Nevertheless, rotenone-induced gut microbiota changes observed in our study are similar to the gut microbiota composition in PD patients.^27–29^ While these results further support a role for gut microbiota in PD, whether these specific subsets of microbial taxa are responsible for driving PD pathophysiology needs to be confirmed in future studies.

In conclusion, our study highlights a potentially important role for gut microbiota as a determinant of motor dysfunction in PD. It paves the way for future investigation on how specific bacterial strains and metabolites may play a role in regulating CNS processes that contribute to motor dysfunction.

## Materials and methods

### Mice

Swiss Webster germ-free (GF) male and female mice aged 10–12 weeks were housed in a flexible film isolator in the Mayo Clinic gnotobiotic facility.^30,31^ CR age/gender-matched Swiss Webster mice purchased from Taconic Farms. Mice were gavaged every day for 6 weeks with rotenone or vehicle as described in the protocol below. At the end of the sixth week, motor function was assessed and an *in-vivo* permeability assay was performed. Mice were then euthanized followed by a collection of brain and colon samples for IHC and *ex-vivo* permeability studies. Pellet samples were collected and stored at −80°C for 16S microbiota analysis. All mice studies were approved by the Mayo Clinic Institutional Animal Care and Use Committee and conducted in compliance with regulatory guidelines.

### Gavage protocol

Rotenone solution was prepared by dissolving 10 mg/kg of rotenone (Tocris. Bio-Techne, Minneapolis, MN, USA) in a vehicle solution containing 4% carboxymethylcellulose and 1.25% chloroform. Age and weight-matched CR mice were randomly assigned to be gavaged every day either with freshly prepared rotenone solution (rotenone group) or vehicle solution (control group) for 6 weeks. GF mice were treated similarly with daily gavage, but sufficient volume of a freshly prepared batch of rotenone and vehicle only solution were made every 3–4 days under sterile conditions, and transferred into the isolators to reduce chances of contamination.

### Quantification of tyrosine hydroxylase (TH) neurons

Loss of tyrosine hydroxylase (TH) motor neurons in substantia nigra (SN) was evaluated using immunohistochemistry (IHC). Briefly, brain samples from rotenone and vehicle solution administered mice were fixed in 4% paraformaldehyde for 48–72 h followed by 24–48 h of cryoprotection with 30% sucrose solution. Midbrain sections (~3 mm) were collected using Zivic mouse brain slicer (Zivic instruments, Pittsburgh, USA) and embedded in Tissue-Tek® OCT. freezing medium for cryostat sectioning. About 40 µm brain slices were washed 3X with 1× PBS solution at 10 min interval. Sections were blocked for 4 h at room temperature in 10% normal donkey serum (NDS) containing 0.6% Triton X-100 in 1× PBS. After 4 hours, rabbit anti-tyrosine hydroxylase (TH; Abcam ab75875 1:600) was added in a blocking serum containing 2% NDS + 0.1% Triton X-100 PBS. Sections were incubated overnight at 4°C and the primary antibody was removed and washed (3X; 15 min/wash). After washing, sections were incubated in secondary antibody solution containing donkey anti-rabbit FITC (Abcam ab6798; 1:400) in blocking serum for 4 h at room temperature shielded from light. The tissues were finally rinsed 3X with 1× PBS solution and mounted on slides with DAPI mounting medium followed by coverslips. Brain slices were analyzed using a fluorescent microscope (Leica microsystems, Leica Application suite X v 3.6.0).

### Measurement of grip strength and motor coordination

***Grip strength test*** was performed to evaluate forelimb motor strength using a grip strength meter (Chatillon, Columbus Instruments, Columbus, USA). The grip strength measurement mode was set to peak tension mode and units set to pounds-force (lbf). The mice were then gently removed from the cage and allowed to grip the metal bar tightly with both paws. Mice were slowly pulled away from the metal bar (or grid) in a straight horizontal line until their grip was broken and the peak force was recorded. This step was repeated 3 times without resting. After the third round of force measurement, mice were put back in their cages and allowed to rest for at least a minute. The entire test was repeated 5 times for a total of 15 pulls/mice. The grip strength was then averaged for the final analysis.

***Rotarod test*** was used to examine motor coordination and sensory-motor function in mice. Mice were briefly trained and screened by placing mice on the rod at a fixed speed of 10 and 20 RPM. All test mice were screened for their ability to remain on the bar for at least 5 sec at 10 and 20 RPM. Mice were allowed at least three trials, and if a mouse was unable to stay in the bar for more than 5 sec during trials, the mouse was excluded from the study. After initial screening, the rotarod was used in an accelerating ramp mode with a minimum acceleration of 4 RPM and a maximum acceleration of 40 RPM with a ramp duration of 4 min. The time duration that the mice were able to remain on the bar was recorded. The process was repeated 3 times with a 2 min interval between each trial.

### Ex-vivo intestinal permeability measurement

Proximal colon sections were obtained from rotenone and vehicle administered GF and CR mice. The colonic luminal contents were then quickly but gently flushed with chilled Krebs solution (composition in mM/l: 11.5 d-glucose/d-mannitol, 120.3 NaCl, 15.5 NaHCO3, 5.9 KCl, 1.2 NaH2PO4, 2.5 CaCl2 · 2H2O, and 1.2 MgCl2; pH 7.3–7.4) using a 30 ml syringe hooked to a 200 µl pipette tip. Tissues were then opened along the mesenteric border, flipped, and pinned flat on the Sylgard with mucosa side facing down. The underlying muscle layer was peeled away under a stereomicroscope with fine forceps and mucosa-submucosa preparations were mounted on Ussing chamber cassette. A 4 ml aliquot of 4 mg/ml of FITC (Fluorescein isothiocyanate 4 KDa; Sigma–Aldrich Catalog 60842–46-8), a well-characterized paracellular diffusion marker, was added to the mucosal side, and samples were obtained from the serosal side at regular intervals over a period of 3 h. The cumulative amount of FITC fluorescence accumulated in the serosal samples was assayed and plotted against time. The slope of the linear portion of the cumulative amount versus time profile provides an estimate of FITC flux across the mucosal explants.

### In-vivo permeability measurement

*In vivo* permeability was measured using the method described previously.^32^ Briefly, on the day of measurement mice were fasted for 2 h then given a 200 µl oral gavage containing 100 mg/mL creatinine, 60 mg/mL FITC-4 kDa Dextran, and 40 mg/mL of rhodamine B isothiocyanate-70 kDa dextran (Sigma) to allow assessment of pore (radius 2.6 Å), leak (radius 13 Å) and unrestricted (radius 64 Å) barrier pathways, respectively.^32^ Mice were fasted for additional 2 h after which food was replaced. Five hours post-gavage, the intracardiac puncture was performed to harvest blood and collect serum samples. Serum samples were sent to the Mayo Clinic Immunochemical core for creatinine analysis while fluorescence for FITC and RITC was measured using a Synergy Mx (BioTek) at excitation wavelengths of 485 and 553 nm and emission wavelengths of 528 and 627 nm respectively. These were converted into absolute concentrations using standard curves.

### 16S rRNA amplicon-based sequencing and data analysis

Bacterial DNA was extracted from a stool after bead beating using the MoBio fecal DNA extraction kit (Mo Bio Laboratories, Carlsbad, CA, USA), followed by 16S ribosomal ribonucleic acid (rRNA) amplification using Nextera library compatible primers flanking the V4 hypervariable region ([forward overhang] +515 F: [TCGTCGGCAGCGTCAGATGTGTATAAGAGACAG]GTGCCAGCMGCCGCGGTAA; and [reverse overhang] +806 R: [GTCTCGTGGGCTCGGAGATGTGTATAAGAGACAG]GGACTACHVGGGTWTCTAAT) and prepared for sequencing using a dual-indexing protocol. All samples were sequenced together in 2 × 300 paired-end mode on an Illumina MiSeq instrument by the University of Minnesota Genomics Center. All sequences and metadata are available at the short-read archive (Accession number: PRJNA673724).

Microbiome diversity analyses was done using QIIME 2 v2020.2,^33^ while other data analyses, statistical tests, and visualizations were performed in R. Differences in alpha diversity were assessed based on Faith’s Phylogenetic Diversity metric. The Kruskal–Wallis (pairwise) test was used to test differences in alpha diversity between groups and within groups at baseline and week 6. Differences in beta diversity were computed based on the Bray Curtis metric and statistical significance between groups at each time point was assessed using PERMANOVA. Comparison of change in alpha or beta diversity from baseline to week 6 following treatment of CR mice with vehicle or rotenone was done in R using Wilcoxon Sign–Rank Test. The operational taxonomic unit (OTU) table for all samples generated in QIIME 2 was used to assess differences in the relative abundance of microbial genera using the DEseq2 package in R. Predictive functional profiling from 16S rRNA was performed using the Phylogenetic Investigation of Communities by Reconstruction of Unobserved States (PICRUSt2)^34^ and significant differences (FDR <0.25) among the groups are shown.

## References

[1] DeMaagd G, Philip A. Parkinson’s disease and its management: part 1: disease entity, risk factors, pathophysiology, clinical presentation, and diagnosis. P T. 2015;40:504–9. 26236139 PMC4517533

[2] Marras C, Beck JC, Bower JH, Roberts E, Ritz B, Ross GW, Abbott RD, Savica R, Van Den Eeden SK, Willis AW, et al. Prevalence of Parkinson’s disease across North America. NPJ Parkinsons Dis. 2018;4(1):21. doi:10.1038/s41531-018-0058-0. 30003140 PMC6039505

[3] Power JH, Barnes OL, Chegini F. Lewy bodies and the mechanisms of neuronal cell death in Parkinson’s disease and dementia with lewy bodies. Brain Pathol. 2017;27(1):3–12. doi:10.1111/bpa.12344. 26667592 PMC8029402

[4] Bereczki D. The description of all four cardinal signs of Parkinson’s disease in a Hungarian medical text published in 1690. Parkinsonism Relat Disord. 2010;16(4):290–293. doi:10.1016/j.parkreldis.2009.11.006. 19948422

[5] Wu SL, Liscic RM, Kim S, Sorbi S, Yang YH. Nonmotor symptoms of Parkinson’s disease. Parkinsons Dis. 2017;2017:4382518. 28352489 10.1155/2017/4382518 PMC5352894

[6] Poirier AA, Aubé B, Côté M, Morin N, Di Paolo T, Soulet D. Gastrointestinal dysfunctions in Parkinson’s disease: symptoms and treatments. Parkinsons Dis. 2016;2016:6762528. doi:10.1155/2016/6762528. 28050310 PMC5168460

[7] Makaroff L, Gunn A, Gervasoni C, Richy F. Gastrointestinal disorders in Parkinson’s disease: prevalence and health outcomes in a US claims database. J Parkinsons Dis. 2011;1(1):65–74. doi:10.3233/JPD-2011-001. 23939257

[8] Abbott RD, Petrovitch H, White LR, Masaki KH, Tanner CM, Curb JD, Grandinetti A, Blanchette PL, Popper JS, Ross GW, et al. Frequency of bowel movements and the future risk of Parkinson’s disease. Neurology. 2001;57(3):456–462. doi:10.1212/WNL.57.3.456. 11502913

[9] Bhattarai Y, Kashyap PC. Parkinson’s disease: are gut microbes involved? Am J Physiol Gastrointest Liver Physiol. 2020. 10.1152/ajpgi.00058.2020 PMC8087343 32877215

[10] Dawson TM, Ko HS, Dawson VL. Genetic animal models of Parkinson’s disease. Neuron. 2010;66(5):646–661. doi:10.1016/j.neuron.2010.04.034. 20547124 PMC2917798

[11] Hatcher JM, Pennell KD, Miller GW. Parkinson’s disease and pesticides: a toxicological perspective. Trends Pharmacol Sci. 2008;29(6):322–329. doi:10.1016/j.tips.2008.03.007. 18453001 PMC5683846

[12] Miyazaki I, Isooka N, Imafuku F, Sun J, Kikuoka R, Furukawa C, Asanuma M. Chronic systemic exposure to low-dose rotenone induced central and peripheral neuropathology and motor deficits in mice: reproducible animal model of Parkinson’s disease. Int J Mol Sci. 2020;21(9):3254. doi:10.3390/ijms21093254. PMC7246801 32375371

[13] Cannon JR, Tapias V, Na HM, Honick AS, Drolet RE, Greenamyre JT. A highly reproducible rotenone model of Parkinson’s disease. Neurobiol Dis. 2009;34(2):279–290. doi:10.1016/j.nbd.2009.01.016. 19385059 PMC2757935

[14] Barichella M, Severgnini M, Cilia R, Cassani E, Bolliri C, Caronni S, Ferri V, Cancello R, Ceccarani C, Faierman S, et al. Unraveling gut microbiota in Parkinson’s disease and atypical parkinsonism. Mov Disord. 2019;34(3):396–405. doi:10.1002/mds.27581. 30576008

[15] Golden JP, Demaro JA, Knoten A, Hoshi M, Pehek E, Johnson EM, Gereau RW, Jain S. Dopamine-dependent compensation maintains motor behavior in mice with developmental ablation of dopaminergic neurons. J Neurosci. 2013;33(43):17095–17107. doi:10.1523/JNEUROSCI.0890-13.2013. 24155314 PMC3807031

[16] Welchko RM, Lévêque XT, Dunbar GL. Genetic rat models of Parkinson’s disease. Parkinsons Dis. 2012;2012:128356. doi:10.1155/2012/128356. 22550609 PMC3328158

[17] Haugarvoll K, Bindoff LA, Tzoulis C. Nigrostriatal denervation sine parkinsonism. Brain. 2016;139(Pt 4):e25. doi:10.1093/brain/awv410. 26811251

[18] Schöls L, Reimold M, Seidel K, Globas C, Brockmann K, Karsten Hauser T, Auburger G, Bürk K, den Dunnen W, Reischl G, et al. No parkinsonism in SCA2 and SCA3 despite severe neurodegeneration of the dopaminergic substantia nigra. Brain. 2015;138(Pt 11):3316–3326. doi:10.1093/brain/awv255. 26362908

[19] Sampson TR, Debelius JW, Thron T, Janssen S, Shastri GG, Ilhan ZE, Challis C, Schretter CE, Rocha S, Gradinaru V, et al. Gut microbiota regulate motor deficits and neuroinflammation in a model of Parkinson’s disease. Cell. 2016;167(6):1469–1480.e1412. doi:10.1016/j.cell.2016.11.018. 27912057 PMC5718049

[20] Sanchez S, Rodríguez-Sanoja R, Ramos A, Demain AL. Our microbes not only produce antibiotics, they also overproduce amino acids. J Antibiot (Tokyo). 2017. doi:10.1038/ja.2017.142. 29089597

[21] Baj A, Moro E, Bistoletti M, Orlandi V, Crema F, Giaroni C. Glutamatergic signaling along the microbiota-gut-brain axis. Int J Mol Sci. 2019;20(6). doi:10.3390/ijms20061482. PMC6471396 30934533

[22] Strandwitz P. Neurotransmitter modulation by the gut microbiota. Brain Res. 2018;1693(Pt B):128–133. doi:10.1016/j.brainres.2018.03.015. 29903615 PMC6005194

[23] Barrett E, Ross RP, O’Toole PW, Fitzgerald GF, Stanton C. γ-Aminobutyric acid production by culturable bacteria from the human intestine. J Appl Microbiol. 2012;113(2):411–417. doi:10.1111/j.1365-2672.2012.05344.x. 22612585

[24] Pokusaeva K, Johnson C, Luk B, Uribe G, Fu Y, Oezguen N, Matsunami RK, Lugo M, Major A, Mori-Akiyama Y, et al. GABA-producing Bifidobacterium dentium modulates visceral sensitivity in the intestine. Neurogastroenterol Motil. 2017;29(1):e12904. doi:10.1111/nmo.12904. PMC5195897 27458085

[25] Perez-Pardo P, Dodiya HB, Engen PA, Naqib A, Forsyth CB, Green SJ, Garssen J, Keshavarzian A, Kraneveld AD. Gut bacterial composition in a mouse model of Parkinson’s disease. Benef Microbes. 2018;9(5):799–814. doi:10.3920/BM2017.0202. 30099890

[26] Johnson ME, Stringer A, Bobrovskaya L. Rotenone induces gastrointestinal pathology and microbiota alterations in a rat model of Parkinson’s disease. Neurotoxicology. 2018;65:174–185. doi:10.1016/j.neuro.2018.02.013. 29471018

[27] Scheperjans F, Aho V, Pereira PA, Koskinen K, Paulin L, Pekkonen E, Haapaniemi E, Kaakkola S, Eerola‐Rautio J, Pohja M, et al. Gut microbiota are related to Parkinson’s disease and clinical phenotype. Mov Disord. 2015;30(3):350–358. doi:10.1002/mds.26069. 25476529

[28] Petrov VA, Saltykova IV, Zhukova IA, Alifirova VM, Zhukova NG, Dorofeeva YB, Tyakht AV, Kovarsky BA, Alekseev DG, Kostryukova ES, et al. Analysis of gut microbiota in patients with Parkinson’s disease. Bull Exp Biol Med. 2017;162(6):734–737. doi:10.1007/s10517-017-3700-7. 28429209

[29] Gerhardt S, Mohajeri MH. Changes of colonic bacterial composition in Parkinson’s Disease and other neurodegenerative diseases. Nutrients. 2018;10(6). doi:10.3390/nu10060708. PMC6024871 29857583

[30] Bhattarai Y, Kashyap PC. Germ-free mice model for studying host-microbial interactions. Methods Mol Biol. 2016;1438:123–135. 27150088 10.1007/978-1-4939-3661-8_8

[31] Bhattarai Y, Williams BB, Battaglioli EJ, Whitaker WR, Till L, Grover M, Linden DR, Akiba Y, Kandimalla KK, Zachos NC, et al. Gut microbiota-produced tryptamine activates an epithelial G-protein-coupled receptor to increase colonic secretion. Cell Host Microbe. 2018;23(6):775–785.e775. doi:10.1016/j.chom.2018.05.004. 29902441 PMC6055526

[32] Edogawa S, Edwinson AL, Peters SA, Chikkamenahalli LL, Sundt W, Graves S, Gurunathan SV, Breen-Lyles M, Johnson S, Dyer R, et al. Serine proteases as luminal mediators of intestinal barrier dysfunction and symptom severity in IBS. Gut. 2020;69(1):62–73. doi:10.1136/gutjnl-2018-317416. 30923071 PMC6765451

[33] Bokulich NA, Kaehler BD, Rideout JR, Dillon M, Bolyen E, Knight R, Huttley GA, Gregory Caporaso J. Optimizing taxonomic classification of marker-gene amplicon sequences with QIIME 2’s q2-feature-classifier plugin. Microbiome. 2018;6(1):90. doi:10.1186/s40168-018-0470-z. 29773078 PMC5956843

[34] Douglas GM, Maffei VJ, Zaneveld JR, Yurgel SN, Brown JR, Taylor CM, Huttenhower C, Langille MG. PICRUSt2 for prediction of metagenome functions. Nat Biotechnol. 2020;38(6):685–688. doi:10.1038/s41587-020-0548-6. 32483366 PMC7365738

